# Urogenitale Tumoren nach Nierentransplantation – monozentrische Aufarbeitung der Inzidenzen und Überblick urologischer Vorsorgemaßnahmen

**DOI:** 10.1007/s00120-024-02317-3

**Published:** 2024-03-21

**Authors:** J. Putz, V. Kestel, R. Herout, A. Borkowetz, S. Leike, C. Thomas, M. Baunacke

**Affiliations:** 1 Klinik und Poliklinik für Urologie, Universitätsklinikum Carl Gustav Carus an der Technischen Universität Dresden, Fetscherstr. 74, 01307 Dresden, Deutschland; 2 Medizinische Fakultät, Universitätsklinikum Carl Gustav Carus der Technischen Universität Dresden, Dresden, Deutschland

**Keywords:** Nierentransplantation, Urogenitale Tumoren, Nierenzellkarzinom, Risikofaktoren, Nachsorge, Kidney transplantation, Urogenital tumours, Renal cell carcinoma, Risk factors, Aftercare

## Abstract

**Hintergrund:**

Urogenitale Tumoren zählen zur den häufigsten soliden Malignomen nach Nierentransplantation (NTX).

**Fragestellung:**

Es erfolgte die Erfassung von Inzidenz und Mortalität urogenitaler Tumoren nach NTX im eigenen Patientengut und die Übertragung der Erkenntnisse in Bezug auf empfohlene Nachsorgenotwendigkeit und -frequenz.

**Material und Methode:**

Durchführung einer retrospektiven monozentrischen Erfassung von Tumorerkrankungen allgemein und Urogenitaltumoren spezifisch von Patienten, die zwischen 2010 bis 2020 eine Nierentransplantation am Transplantationszentrum Dresden erhalten haben. Daraus wurden Handlungsempfehlungen für die Praxis als Nachsorgekonzept abgeleitet.

**Ergebnisse:**

Insgesamt 13 % (93/710) der nierentransplantierten Patienten entwickelten eine Neoplasie. Patienten mit einem höheren Alter (60,1 ± 10,6 vs. 53,8 ± 12,5 Jahre; *p* < 0,001), einem erhöhten Charlson-Score (≥ 4: 68 % vs. 46 %; *p* < 0,001) und einer früheren Tumoranamnese (18 % vs. 8 %; *p* < 0,001) wiesen häufiger eine Tumordiagnose nach Transplantation auf. In der multivariaten Analyse zeigte sich dabei die frühere Tumoranamnese als unabhängiger Prädiktor für eine Tumorentwicklung nach Transplantation (OR 2,2; 95 %-KI [1,2–4,1]; *p* = 0,01). Von allen Tumorerkrankungen entfielen 30 % (28/93) auf urogenitale Tumoren. Am häufigsten dabei war die Entwicklung eines Nierenzellkarzinoms der Nativnieren (*n* = 12), am zweithäufigsten Prostatakarzinome (*n* = 9).

**Schlussfolgerung:**

Urogenitale Tumoren bilden einen Großteil solider Malignome nach NTX. Aufgrund der Häufigkeit besteht die dringende Notwendigkeit einer dauerhaften Nachsorge sowie der spezialisierten urologischen Therapie. Bereits vor Listung zur Transplantation können Risikofaktoren erkannt und individuelle Konzepte zur Nachbetreuung erstellt werden.

**Graphic abstract:**

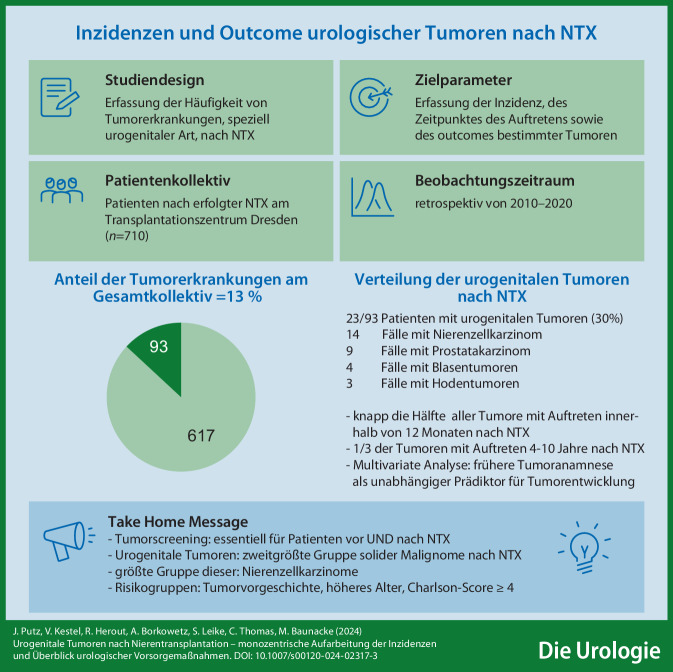

## Abstract (en)

**Background:**

Urogenital tumors are among the most common solid malignancies after kidney transplantation (TX).

**Objective:**

We analyzed the incidence and mortality of urogenital tumors after kidney TX in our own patient population as well as answered the question of recommended follow-up necessity and frequency in this cohort.

**Materials and methods:**

Retrospective monocentric data collection of tumor diseases and the most common urogenital tumors after kidney TX at the Transplant Center Dresden between 2010 and 2020 was done. From this, we derived recommendations for a useful follow-up concept.

**Results:**

A total of 13% (93/710) of kidney TX patients developed a neoplasm. Older patients (60.1 ± 10.6 vs. 53.8 ± 12.5; *p* < 0.001), with higher Charlson scores (≥ 4: 68% vs. 46%; *p* < 0.001) and a previous tumor history (18% vs. 8%; *p* < 0.001) were more likely to develop a neoplasm after transplantation. In the multivariate analysis, previous tumor history was found to be an independent predictor of tumor development after renal transplantation (OR 2.2; 95%-KI [1.2–4.1]; *p* = 0.01). Urogenital tumors accounted for 30% (28/93) of all malignancies. Renal cell carcinoma of the native kidney was the most common (*n* = 12) neoplasm, followed by prostate cancer (*n* = 9).

**Conclusion:**

Most solid malignancies after kidney TX arise from the urinary tract. Due to their frequency, there is an urgent need for specialized urological therapy and long-term follow-up care. Even before listing for TX, risk factors can be recognized and individual concepts for follow-up care can be developed.

## Einleitung

Tumoren nach Nierentransplantation (NTX) zählen zu den häufigsten Todesursachen betroffener Patienten. Dabei bilden urogenitale Tumoren die zweitgrößte Gruppe solider Malignome. Nach Hauttumoren als den geläufigsten Neoplasien gehören Nierenzell(NCC)- und Urothelkarzinome (BC), Prostatatumoren (PCa) oder auch Tumoren des äußeren Genitales zur großen Gruppe weiterer Erkrankungen, die einer besonderen Kontrolle von Risikopatienten unter Immunsuppression bedürfen. Die folgende Originalarbeit beschäftigt sich mit der Häufigkeit wesentlicher urogenitaler Tumoren im eigenen Patientengut und leitet daraus die Relevanz engmaschiger urologischer Nachsorge ab.

## Hintergrund

Patienten nach NTX sind aufgrund der dauerhaft erforderlichen Immunsuppression einem allgemein erhöhten Tumorrisiko ausgesetzt. Die Ursachen sind multifaktoriell, nämlich die Exposition gegenüber den Immunsuppressiva, aber auch virale Faktoren sowie die Grunderkrankung selbst [3, 4, 8, 25].

So ist beispielsweise das Risiko zur Entwicklung von Tumoren der Eigennieren allein aufgrund der Dialysepflicht und -dauer als solchen gegenüber der gesunden Allgemeinbevölkerung um das 5‑ bis 15-Fache erhöht [8, 22, 24].

Die häufigste Tumorerkrankung nach NTX stellen nicht melanozytäre Hauttumoren dar. Mit bis zu 40 % bilden diese die größte Gruppe maligner Neoplasien innerhalb dieses Patientenguts [19]. Eine Empfehlung der gezielten dermatologischen Nachsorge nach NTX wird daher explizit an alle Patienten formuliert.

Die Kenntnis weiterer Neoplasien bezieht sich häufig auf die sog. Posttransplantationslymphome (PTLD), ausgehend von den lymphatischen Zellen des Immunsystems. Hier liegt die Häufigkeit bei etwa 1–7 % nach NTX bzw. bei 4,5 Fällen pro 1000 Patientenjahren [14].

Assoziationen bestehen dabei in bis zu 70–90 % in EBV(Epstein-Barr-Virus)-positivem Patientengut, weshalb auch hier engmaschige Nachsorgen bei den Patienten durchgeführt werden.

Statistisch gesehen finden sich – unabhängig von einer Transplantation – urogenitale Tumoren unter den häufigsten 15 der Krebsneuerkrankungen in Deutschland. Dazu gehört das PCa (Platz 1 bei Männern), das NCC (Platz 8 bei Männern und Platz 11 bei Frauen) sowie das BC (Platz 4 bei Männern und Platz 13 bei Frauen; [28]).

Betrachtet man insbesondere die soliden Neoplasien nach NTX, so zeigen sich urogenitale Tumoren hier als sehr häufige Malignome mit bis zu 5fach erhöhter Inzidenz, welche im Verlauf auftreten können [15]. Damit stellt die Patientengruppe Nierentransplantierter nicht nur generell, sondern auch für die urologische Tumordiagnostik- und -therapie eine wesentliche Risikogruppe dar, auf welche besonderes Augenmerk im Hinblick auf diese Tumoren und deren qualifizierte Behandlung gerichtet werden sollte.

## Ziele der Arbeit

Kenntnisse über Inzidenz und Mortalität urogenitaler Tumoren nach NTX im eigenen Patientengut am Transplantationszentrum Dresden sollen zur Sensibilisierung auf diesem Gebiet beitragen und damit die Optionen einer möglichst kurativen Therapie durch frühzeitige Detektion dieser Tumoren aufzeigen.

Aus der Häufigkeit des Auftretens von bestimmten Neoplasien soll die Frage nach der empfohlenen urologisch-fachspezifischen Nachsorgenotwendigkeit und -frequenz abgeleitet werden.

## Material und Methode

Im Rahmen einer monozentrischen, retrospektiven Datenerfassung wurden die am Transplantationszentrum Dresden nierentransplantierten Patienten in einem 10-Jahres-Zeitraum (2010 bis 2020) analysiert. Das Gesamtkollektiv wurde auf das Auftreten von Malignomen nach erfolgter Transplantation untersucht. Urogenitale Tumoren wurden differenzierter ausgewertet. In Ergänzung zu allgemeinen demographischen Daten wie Alter, Geschlecht, BMI und Wartezeit wurden Komorbiditäten, der Charlson-Komorbiditätsindex (Charlson-Score), eine Herzinsuffizienz über die NYHA(New York Heart Association)-Einteilung sowie die Abschätzung des perioperativen Risikos über den ASA (American Society of Anesthesiologists) Score erhoben.

Es wurden weiterhin Tumorerkrankungen in der Vorgeschichte, Vortransplantationen, die Immunsuppression und das Auftreten von therapiepflichtigen Abstoßungsreaktionen im primären stationären Aufenthalt untersucht. Für alle Tumoren wurde der Zeitpunkt des Auftretens nach NTX und ein etwaiger Tod durch diese aufgeschlüsselt. Basalzellkarzinome der Haut wurden aufgrund der guten kurativen Therapie mit minimaler Metastasierungsgefahr in der weiteren Auswertung nicht inkludiert. Bei urogenitalen Tumoren wurde spezifisch die Therapie und das Outcome erhoben.

Urogenitale Tumoren wurden wie folgt aufgegliedert: PCa, NCC, BC sowie Hodentumoren (TC). Die statistische Auswertung erfolgte mit t‑Test, χ^2^-Test sowie uni- und multivariater binärer logistischer Regression. Das Signifikanzniveau wurde als *p* < 0,05 festgelegt. Die Berechnungen erfolgten mit „IBM SPSS Statistics 29“ (Armonk, NY, USA). Die Studie entspricht der Deklaration von Helsinki und wurde durch die lokale Ethikkommission genehmigt (BO-EK-15012021).

## Ergebnisse

### Patientenkohorte

Das Gesamtkollektiv der Untersuchung zwischen 2010 und 2020 betrug 710 Patienten, welche in dieser Zeit nierentransplaniert worden waren. Das Follow-up betrug 59,3 ± 36,3 (Range 0–173) Monate. Davon hatten 166 Patienten (23,4 %) im Rahmen einer Lebendnierenspende und 544 Patienten (76,6 %) im Rahmen eines postmortalen Angebots ihr Organ erhalten. Von diesen waren 437 (61,5 %) männliche und 273 (38,5 %) weibliche Empfänger. Das Alter betrug im Mittel 54,6 (Median 56; SD 12,5; Range 18–82) Jahre. Die mittlere Wartezeit auf das transplantierte Organ lag bei 64,3 (Median 58; SD 45,7; Range 0–305) Monaten. Der überwiegende Teil der Patienten hatte eine Ersttransplantation (88,2 %), gefolgt von 76 Zweittransplantierten (10,7 %) und um 1 % Dritttransplantierter. Ein Charlson-Score von ≥ 4 wurde bei 348 der 710 Patienten (49 %) ermittelt. Hinsichtlich der immunsuppressiven Therapie erhielten 560 (78,9 %) Patienten eine Induktionstherapie mit Basiliximab, 646 (90,9 %) Patienten Tacrolimus, 56 (8,4 %) Patienten Cyclosporin A, 666 (93,8 %) Patienten Mycophenolsäure und 62 (8,7 %) Patienten Thymoglobulin. Eine akute Rejektion im primären stationären Aufenthalt hatten nachweislich 84 (11,8 %) Patienten. Eine Tumoranamnese vor der Transplantation wiesen 64 Patienten (9 %) auf (Tab. 1).

**Table 1:** 

Variable		Gesamtkollektiv *n* = 710 (100 %)	Keine Tumorerkrankung nach NTX *n* = 617 (87 %)	Tumorerkrankung nach NTX *n* = 93 (13 %)	*p*-Wert
Alter (Jahre)		54,6 ± 12,5 56 (18–82)	53,8 ± 12,5 55 (19–82)	60,1 ± 10,6 62 (18–77)	*<* *0,001*
Geschlecht	Männlich	437 (62 %)	378 (61 %)	59 (63 %)	0,7
	Weiblich	273 (38 %)	239 (39 %)	34 (37 %)	
BMI (kg/m^2^)		26,4 ± 4,5 25,9 (15,8–42,2)	26,3 ± 4,4 25,8 (15,8–42,2)	27,1 ± 4,5 26,5 (17,6–37,2)	0,1
Diabetes mellitus (*n* = 708)	Ja	134 (19 %)	113 (18 %)	21 (23 %)	0,3
	Nein	574 (81 %)	502 (82 %)	72 (77 %)	
Wartezeit bis NTX (Monate)		64,3 ± 45,7 58 (0–305)	65,1 ± 46,0 59 (0–305)	59,1 ± 43,2 51 (0–211)	0,2
Lebendspende	Ja	166 (23 %)	149 (24 %)	17 (18 %)	0,2
	Nein	544 (77 %)	468 (76 %)	76 (82 %)	
*Komorbidität*					
Charlson-Score	2–3	362 (51 %)	332 (54 %)	30 (32 %)	*<* *0,001*
	≥ 4	348 (49 %)	285 (66 %)	63 (68 %)	
Restdiurese (ml/24 h)		561,8 ± 726,5 200 (0–3.000)	548,6 ± 717,4 150 (0–3.000)	648,3 ± 782,7 250 (0–3.000)	0,2
ASA (*n* = 705)	3	679 (96 %)	590 (96 %)	89 (96 %)	0,7
	4	26 (4 %)	22 (4 %)	4 (4 %)	
NYHA (*n* = 701)	1	624 (88 %)	542 (88 %)	82 (88 %)	0,8
	2	66 (9 %)	56 (9 %)	10 (11 %)	
	3	11 (2 %)	10 (2 %)	1 (1 %)	
Anzahl der NTX	1	626 (88 %)	540 (88 %)	86 (93 %)	0,3
	2	76 (11 %)	69 (11 %)	7 (7 %)	
	3	8 (1 %)	8 (1 %)	0 (0 %)	
Tumoranamnese vor NTX	Ja	64 (9 %)	47 (8 %)	17 (18 %)	*<* *0,001*
	Nein	646 (91 %)	570 (92 %)	76 (82 %)	
*Immunsuppression*					
Cyclosporin A (*n* = 705)	Ja	56 (8 %)	45 (7 %)	11 (12 %)	0,1
	Nein	649 (92 %)	568 (93 %)	81 (88 %)	
Tacrolimus (*n* = 705)	Ja	646 (91 %)	564 (91 %)	82 (88 %)	0,2
	Nein	59 (9 %)	48 (9 %)	11 (12 %)	
Mycophenolsäure (*n* = 700)	Ja	666 (94 %)	576 (93 %)	90 (97 %)	0,2
	Nein	34 (6 %)	32 (7 %)	2 (3 %)	
Basiliximab (*n* = 690)	Ja	560 (79 %)	485 (79 %)	75 (80 %)	0,7
	Nein	130 (21 %)	114 (21 %)	16 (20 %)	
Thymoglobulin	Ja	62 (9 %)	56 (9 %)	6 (6 %)	0,4
	Nein	648 (91 %)	561 (91 %)	87 (94 %)	
Akute therapiebedürftige Abstoßung im primären Aufenthalt (*n* = 709)	Ja	84 (12 %)	72 (12 %)	12 (13 %)	0,7
	Nein	625 (88 %)	544 (88 %)	81 (87 %)	

*NTX* Nierentransplantation, *NYHA* New York Heart Association, *ASA* American Society of Anesthesiologists, *BMI* Body Mass Index; Angaben teils als Mittelwert mit Standardabweichung, Median und Range, teils als prozentuale Anteile. Kursivierte Werte zeigen die jeweils signifikanten *p*-Werte an

### Verteilung der Tumoren nach NTX allgemein

Insgesamt wurden bei 93 Patienten (13 %) Tumoren nach NTX detektiert. Basalzellkarzinome wurden in die Auswertung nicht mit einbezogen (*n* = 45). Am häufigsten traten Hauttumoren auf (*n* = 41; ohne Basalzellkarzinome) und am zweithäufigsten urogenitale Tumoren (*n* = 28; Tab. 2). Patienten mit einem höheren Alter (60,1 ± 10,6 vs. 53,8 ± 12,5 Jahre; *p* < 0,001), einem höheren Charlson-Score (≥ 4: 68 % vs. 46 %; *p* < 0,001) und einer früheren Tumoranamnese (18 % vs. 8 %; *p* < 0,001) wiesen häufiger eine Tumordiagnose nach Transplantation auf (Tab. 1). In der multivariaten Analyse zeigte sich dabei die frühere Tumoranamnese als unabhängiger Prädiktor für eine Tumorentwicklung nach Transplantation (OR 2,2; 95 %-KI [1,2–4,1]; *p* = 0,01; Tab. 3).

**Table 2:** 

		Gesamtanzahl der jeweiligen Tumoren *n*	Tod durch Tumor *n*
*Urogenitale Tumoren*	NCC	14	4
	PCa	9	0
	BC der Harnblase	4	1
	TC	3	0
*Weitere Tumoren*	Hauttumoren	41	3
	Gastrointestinaltrakt	13	2
	Lymphomen	7	2
	Pulmonale Tumoren	7	4
	Gynäkologische Tumoren	3	0
	Sonstige	7	1 (CUP)
Basalzellkarzinom der Haut		45	0

*CUP* „cancer of unknown primary“, *NCC* Nierenzellkarzinom, *PCa* Prostatakarzinom, *BC* Urothelkarzinom, *TC* Hodentumor

**Table 3:** 

	Univariate Analyse		Multivariate Analyse	
Parameter	OR (95%-KI)	*p*-Wert	OR	*p*-Wert
Alter (> 56 Jahre)	2,5 (1,6–4,1)	*<* *0,001*	1,8 (0,9–3,7)	0,1
Charlson-Score (≥ 4)	2,4 (1,5–3,9)	*<* *0,001*	1,4 (0,7–2,9)	0,3
Positive Tumoranamnese vor NTX	2,7 (1,5–5,0)	*0,001*	2,2 (1,2–4,1)	*0,01*

*OR* Odds Ratio, *KI* Konfidenzintervall, *NTX* Nierentransplantation. Kursivierte Werte zeigen die jeweils signifikanten *p*-Werte an

### Urogenitale Tumoren nach NTX

Urogenitale Tumoren konnten bei 28/93 Tumorpatienten (30 %) nachgewiesen werden. Es traten 14 NCC, 9 PCa, 4 BC sowie 3 TC auf. Zwei Patienten vereinten dabei zwei Tumoren auf sich (einmal PCa und NCC sowie einmal BC und NCC).

Das *NCC *trat in 12 Fällen als Tumor der Nativnieren und in 3 Fällen als Tumor des Transplantats selbst auf. Dabei betrug die mittlere Zeit bis zur Diagnose des Tumors im Bereich der Nativnieren 29,4 ± 27,9 (Median 21; Range 0–91) Monate und für die Tumoren am Transplantat wurden diese unmittelbar bei NTX auffällig. Das mittlere Alter bei Erstdiagnose lag bei 60,9 ± 11,2 (Median 62; Range 39–77) Jahren. Histologisch gliederten sich die Tumoren der Eigennieren in 9 klarzellige NCC, 1 papilläres NCC und 1 erworbenes zystennierenassoziiertes NCC. Von einem Patienten lag der histologische Befund nicht vor (Tab. 4). Im Follow-up kam es zum tumorbedingten Tod in 4 Fällen. Hier lag jeweils ein metastasiertes Tumorleiden bereits bei Erstdiagnose vor. Drei Patienten verstarben innerhalb von 6 Monaten nach Erstdiagnose und ein Patient 51 Monate nach Diagnosestellung. Therapeutisch wurden damals Pazopanib bzw. Everolimus eingesetzt.

**Table 4:** 

	Tumor der Nativnieren (*n* = 12)	Tumor im Transplantat (*n* = 3)
Zeit bis Tumordiagnose (Monate)	29,4 ± 27,9 21 (0–91)	0
Alter bei Tumordiagnose (Jahre)	60,9 ± 11,2 62 (39–77)	
*Histologie*		
Klarzelliges NCC	9	2
Papilläres NCC	1	0
Chromophobes NCC	0	1
Zystennierenassoziiertes NCC	1	0
Unbekannte Histologie	1	0

*NCC* Nierenzellkarzinom; Angaben teils als Mittelwert mit Standardabweichung, Median und Range, teils als absolute Zahlen

Die Tumoren des Transplantates wurden in 2 Fällen intraoperativ als vermeintliche Zysten R0 reseziert und waren im Follow-up bis dato ohne Rezidivnachweis. In einem Fall wurde, bei fehlender Schnittbildgebung des Spenders, unwissentlich ein intrarenaler Tumor des Spenders mit transplantiert und die Niere nach Demarkation des Tumors innerhalb von 14 Tagen umgehend wieder explantiert.

Ein *PCa *wurde in 9 Fällen nach NTX detektiert. Das mittlere Alter bei Erstdiagnose lag bei 62,9 ± 9,3 (Median 65; Range 46–77) Jahren. Der Tumor wurde nach 40,3 ± 46,2 (Median 12; Range 0–127) Monaten diagnostiziert (Tab. 5). Histologisch lag in 8 Fällen ein lokal begrenztes und in einem Fall ein bereits lymphogen metastasiertes PCa vor. Das therapeutische Vorgehen war in 4 Fällen eine kurative radikale Prostatektomie, 1 × Brachytherapie, 1 × TOOKAD-Therapie, 1 × Radiatio, 1 × radikale Prostatektomie mit adjuvanter Radiatio und Hormontherapie. In einem Fall war erfolgte die PCa-Diagnose im Rahmen einer radikalen Zystektomie bei BC. Keiner der Patienten verstarb anhand des Tumors im bisherigen Follow-up.

**Table 5:** 

	Gesamtzahl *n*	Alter bei Tumordiagnose (Jahre)	Zeit bis Tumordiagnose (Monate)	Tumorbedingte Todesfälle *n*
*PCa nach NTX*	9	62,9 ± 9,3 65 (46–77)	40,3 ± 46,2 12 (0–127)	0
*BC nach NTX*	4	64 (52–77)	48 (12–78)	1
*TC nach NTX*	3	42 (33–50)	33,3 (0–72)	0

*PCa* Prostatakarzinom, *BC* Urothelkarzinom, * TC* Hodentumor; Angaben teils als Mittelwert mit Standardabweichung, Median und Range, teils als absolute Zahlen

Die Diagnose eines *BC *nach NTX wurde bei 4 Patienten gestellt. Dies waren ausschließlich BC der Harnblase. Das mittlere Alter bei Erstdiagnose lag hier bei 64 (Range 52–77) Jahren. Der Tumor wurde im Mittel nach 48 (Range 12–78) Monaten diagnostiziert (Tab. 5). Histologisch lagen folgende Tumorstadien je einmal vor: pTa „low grade“, pT1 „high grade“, pT1 „high grade“ mit pTis, pT3 N1 „high grade“. Für den Low-grade-Tumor erfolgte eine transurethrale Blasentumorresektion und in den anderen Fällen jeweils die radikale Zystektomie. In einem Fall schloss sich eine adjuvante Chemotherapie mit Gemcitabine und Cisplatin an. Es kam zu einem tumorbedingten Todesfall nach BC im oben genannten metastasierten Stadium 13 Monate nach Erstdiagnose.

*TC *wurden bei 3 Patienten nach NTX im Follow-up festgestellt. Das mittlere Alter bei Erstdiagnose war 42 (Range 33–50) Jahre. Der Tumor wurde im Mittel nach 33,3 (Range 0–72) Monaten diagnostiziert (Tab. 5). Histologisch lag in allen 3 Fällen ein reines Seminom im klinischen Stadium IA vor, welches mittels Ablatio testis kurativ therapiert wurde.

## Diskussion

Die Untersuchung unseres Patientengutes über 10 Jahre hinweg zeigte eine *Häufigkeit von 13* *% in Bezug auf das Auftreten eines Malignoms nach Nierentransplantation*. In der multivariaten Analyse war das *Vorhandensein eines Tumors in der Voranamnese ein unabhängiger Risikofaktor* für das Vorkommen eines Tumors auch nach Transplantation.

*Urogenitale Tumoren *stellten die *zweithäufigste Gruppe (30* *%) an Tumorerkrankungen* nach Transplantation dar. Knapp die *Hälfte der ermittelten Tumoren* trat *innerhalb von 12 Monaten nach NTX* auf, etwa *ein Drittel noch 4–10 Jahre nach NTX* (Tab. 6).

**Table 6:** 

Auftreten der Tumoren	Anzahl
Im 1. Jahr nach NTX	*n* = 12 (44,5 %)
1–3 Jahre nach NTX	*n* = 6 (22,2 %)
4–10 Jahre nach NTX	*n* = 9 (33,3 %)

Allgemein ist das Tumorrisiko nach Transplantation als hoch einzuschätzen. Registerdaten aus Amerika [3] zeigen eine kumulative Inzidenz von bis zu 10 % nach 5 Jahren und 20 % nach 10 Jahren in Bezug auf das Auftreten mindestens eines Malignoms nach Transplantation. Dies steht im Einklang mit den am hiesigen Zentrum erhobenen Daten. Große Kohortenuntersuchungen aus Australien und Neuseeland konnten ein etwa 4faches oder höheres Risiko für jegliches Malignom nach Transplantation erheben, wobei das Risiko für Nierentumoren etwa 8fach und Blasentumoren etwa 5fach erhöht war [23, 25]. Die häufigsten Tumoren nach NTX sind Hauttumoren, gefolgt von PTLD. Diese lymphatischen Tumoren tragen wesentlich zur Morbidität bei, wobei der überwiegende Anteil der detektierten Hauttumoren kurativ therapierbar ist. In der eigenen Kohorte war der Anteil an Lymphompatienten niedriger als erwartet – in Studien bis 11 % aller NTX-Patienten, hier glücklicherweise nur bei 7,5 %. Möglicherweise ist dies Resultat einer angepassten Immunsuppression an das individuelle Risikoprofil der Patienten für EBV-positive Spender und EBV-negative Empfänger bei allgemein niedrigeren Immunsuppressivaspiegeln als bei anderen soliden Organtransplantationen [12].

Betrachtet man nur die urogenitalen Tumoren im Speziellen, so ist für das *NCC* ein mindestens 7fach erhöhtes Risiko der Tumorentwicklung nach NTX gegenüber der Normalbevölkerung beschrieben [1, 9, 13]. Es ist der Tumor mit dem größten Anteil unter den soliden Tumoren nach NTX. Dies deckt sich auch mit der aktuellen Auswertung (hier 15 % der erfassten Tumoren). Auch das Auftreten in den Nativnieren ist größtenteils kongruent zu unseren Daten. Es gibt Hinweise, wonach Patienten auch je nach Grunderkrankung ein erhöhtes Risiko für die Entstehung eines NCC zeigen könnten. Dies ist v. a. für diabetisch bedingte, hypertensive oder polyzystisch bedingte Nierenkrankheiten beschrieben [26]. Im eigenen Patientengut fand sich bei der Hälfte der Betroffenen eine solche Niereninsuffizienzgenese. Bei funktionierendem Transplantat steht ein kuratives Vorgehen mittels Nephrektomie der tumortragenden Niere als Empfehlung im Vordergrund. Etwa 85 % der Betroffenen können so operativ geheilt werden, da sich zumeist ein T1a-Stadium nachweisen lässt [16]. Empfehlungen einer Listung zur NTX mit Belassen des Nierentumors, auch bei kleinen Befunden, sehen wir als äußerst kritisch an [6]. Vier tumorbedingte Todesfälle, bei denen alle Patienten ein bei Erstdiagnose bereits metastasiertes Leiden hatten, waren in der eigenen Kohorte zu verzeichnen. Sie wurden nach damals aktuellen Therapieschema mit einer Thyrosinkinaseinhibitor- (Pazopanib) sowie mTOR-Inhibitor- (Everolimus) Therapie behandelt. Aktuell stehen auch im metastasierten und lokal fortgeschrittenen Stadium gute Immuntherapeutika, auch in Kombinationen, zur NCC-Therapie zur Verfügung. Gerade für transplantierte Patienten kann hier jedoch der Benefit kaum ausgereizt werden, da bis zu 65 % einer mittels Immuntherapie behandelten Transplantationspatienten (in Studien zumeist Melanome) innerhalb von wenigen Wochen akute Transplantatrejektionen mit Transplantatverlust zeigen [10, 17, 18]. In derartigen Fällen muss im individuellen Gespräch mit dem Patienten ein Entscheid für oder gegen diese Möglichkeit – gerade bei funktionierendem Transplantat – getroffen werden. Ziel sollte es sein, möglichst alle Tumoren im kurativen Setting therapieren zu können. Wesentlich hierfür ist eine gute Evaluation der Patienten von Beginn an. Wie unsere Daten zeigen konnten, ist insbesondere eine Prä-NTX-Tumoranamnese ein signifikanter Prädiktor für das erhöhte Risiko einer Tumorerkrankung nach Transplantation. Wir empfehlen daher ein dezidiertes Screening schon bei Aufnahme auf die Warteliste in Bezug auf ein NCC und auch die unten genannten Tumorentitäten.

Gerade für das NCC ist eine kostengünstige und schnelle Sonographie der Nativnieren Grundbestandteil des Routinescreenings. Dies spiegelt sich auch mit den Empfehlungen der European Best Practice Guidelines for Renal Transplantation und den EAU-Guidelines wider [27, 29]. Im postoperativen Setting ist die mindestens jährliche Kontrolle der Nativnieren – unabhängig vom sicher häufiger untersuchten Transplantat – ebenfalls essentiell. Auch hier können über eine Sonographie kurativ operierbare Tumoren häufig detektiert werden. Dies gilt auch für Schrumpfnieren in der Verlaufsbeurteilung [11]. Für unser Zentrum ist daher eine mindestens jährliche urologische und nephrologische Sonographie Teil der Routine für das Patientengut. Niedergelassene Kollegen erhalten ebenfalls entsprechende Empfehlungen für die außerklinische Nachsorge. Erweiterte Bildgebung mittels Computertomographie oder Magnetresonanztomographie kommen bei schwer einzuschätzenden Befunden oder Vortherapien wie einer Teilresektion der Niere zum Einsatz.

Die Transmission maligner Tumoren am Spenderorgan selbst ist als sehr gering anzusehen (< 0,1 %; [30]). In unserem Fall wurde 2‑mal ein zystischer Befund histologisch untersucht, der sonst ggf. auch als benigne eingeschätzt worden wäre. Damit ist die Anzahl an Tumortransmissionen an der Niere ggf. generell unterschätzt, jedoch zumeist ohne Konsequenz im Langzeitverlauf. Aktuell kann über die zumeist routinemäßigen Spenderbildgebungen ein hohes Maß an Sicherheit beim Empfänger erreicht werden.

Die Prävalenz eines *PCa *unterscheidet sich nahezu nicht vom durchschnittlichen Auftreten in der Normalbevölkerung. In Deutschland gibt es ca. 65.000 Neuerkrankungen pro Jahr [9]. Im eigenen Patientengut waren insgesamt 9,7 % aller Post-NTX Malignompatienten hiervon betroffen. Zum Großteil wurde ein lokal begrenztes PCa diagnostiziert. In der Literatur wird in den meisten Fällen lediglich ein moderat erhöhtes Risiko für ein PCa nach NTX angegeben [20]. Höhere Inzidenzen könnten dabei aus der möglicherweise engmaschiger gescreenten Population nach NTX resultieren, da hier in aller Regel auch eine adäquate urologische Vor- und Nachsorge eine große Rolle spielt. Mittlerweile jedoch können auch Patienten mit lokal begrenzten Niedrigrisiko-PCa umgehend zur NTX gelistet werden. In der very low Situation sogar ohne aktive kurative Therapie [2, 21]. Therapeutisch steht NTX-Patienten die gesamte verfügbare Bandbreite etablierter urologischer Spezialtherapie von der Operation, über die Radiatio oder lokal ablative Verfahren, zur Verfügung. Ziel muss es sein, den Tumor gemäß leitliniengerechter Empfehlung im kurativ intendierten Stadium zu detektieren. Auch hier ist vor einer aktiven NTX-Listung eine Beratung der Patienten gemäß S3-Leitlinie zu fordern, um erstens relevante Karzinome zu selektieren und andererseits die Aufnahme auf die Warteliste nicht grundlos zu verzögern sofern eine geringe Relevanz des Tumors in Bezug auf das Gesamtüberleben vorliegt. Als Nachsorgeempfehlung sprechen wir uns hier für eine jährliche Vorsorge mittels PSA-Screening, rektaler Untersuchung und ggf. erweiterter Bildgebung vor eventueller Biopsie aus.

Risikofaktoren eines *BC *sollten dringend bereits vor Listung zur NTX in der Anamnese dezidiert hinterfragt werden. Gerade ein langjähriger Nikotinabusus muss Anlass zur großzügigen Abklärung des Harntraktes im Falle auffälliger Mikrohämaturie oder rezidivierender Infekte sein.

Bei auch hier hoher genereller Neuerkrankungsrate dieses Tumors i. Allg. (ca. 30.000 Neuerkrankungen pro Jahr in Deutschland [28]), wird das in der Literatur beschriebene Risiko für Transplantierte nochmals auf das 2‑ bis 5Fache angegeben [15]. Therapie der Wahl ist ein zügiges Handeln bei noch lokal begrenztem Tumor. Eine Instillationstherapie der Blase beispielsweise ist bei fehlender Zulassung von BCG unter Immunsuppression in der High-risk-Situation eher kritisch zu betrachten – hier würde unsere Beratungsempfehlung in Richtung einer zeitnahen Zystektomie tendieren. Auch hier sind die Optionen der neuen Immuntherapeutika zwar prinzipiell gegeben, es muss jedoch, wie bereits oben beim NCC ausgeführt, eine kritische Nutzen-Risiko-Abwägung diesbezüglich erfolgen. Metastasierte Stadien können nur über eine sehr enge Nachbetreuung gefährdeter Patienten vermieden werden, da das outcome auch unter Chemotherapie in diesen Fällen sehr schlecht ist [5]. Daher sollte auch in Low-risk-Situationen eine mindestens jährliche Zystoskopie – neben Sonographie sowie Urinkontrolle als Sediment und Zytologie – als gute und wenig invasive Methodik als Nachsorge empfohlen sein. Zudem sollte jede Makrohämaturie, auch bei scheinbar infektassoziierter Genese, großzügig hinterfragt werden.

Zum *TC *ist die Datenlage nach NTX eher begrenzt. Zumeist wird eine ähnliche Inzidenz wie in der Allgemeinbevölkerung angegeben [8]. In der urologischen Vor- und Nachsorge muss die Untersuchung und Sonographie des äußeren Genitales Teil der Routine sein. Nur so können Tumoren im kurativen Stadium erkannt werden. Dies traf für unsere hier detektierten Patienten zu. Bei adäquater Nierenfunktion steht den Patienten auch im metastasierten Stadium die etablierte, platinbasierte Chemotherapie offen. Um dies zu verhindern, sollten Patienten, bei denen eine Risikoanamnese vorliegt (Hodenhochstand, positive Familienanamnese, verkleinerte Hoden; [7]), auch zur regelmäßigen Selbstuntersuchung angehalten werden. Aufklärung und Edukation im Rahmen der Nachsorge ist damit auch Teil der fachspezifischen urologischen Behandlung.

Zusammenfassend kann gesagt werden, dass bei Detektion im frühen Stadium, viele der oben genannten urogenitalen Tumoren so erkannt werden können, dass eine kurative Therapie möglich ist. Dies unterstreichen die Daten unseres Zentrums, wo eine enge urologische Nachsorge Teil der interdisziplinären Betreuung ist. Limitation unserer Untersuchung ist dennoch die retrospektive unizentrische Datenerfassung. Da jedoch die enge Betreuung der meisten Patienten urologisch am TX-Zentrum selbst erfolgt, ist von einer hohen Qualität der erfassten Daten auszugehen, welche die Kohorte Nierentransplantierter sehr gut repräsentiert. Die Tatsache, dass bis zu einem Drittel des Kollektivs das Auftreten des Tumors erst 4 Jahre oder später nach NTX lag, unterstreicht die Empfehlung zur lebenslangen Nachsorge, was im Gegensatz zu einigen Tumornachsorgeempfehlungen für die Allgemeinbevölkerung steht. Die Kenntnis der herausgearbeiteten Risikofaktoren für Patienten, welche zur Listung einer NTX anstehen, ist essentiell, um diesem sensiblen Patientengut bereits beim Erstkontakt die bestmögliche Vorsorge bieten zu können. Ein PSA-Wert allein ersetzt dabei vor als auch nach NTX nicht eine adäquate und umfassende urologische Untersuchung. Eine fachspezifische Aufklärung zum Vorkommen kurativ behandelbarer urogenitaler Tumoren und eine mindestens jährliche fachurologische Vorstellung ist unerlässlich, um ein qualitativ gutes Langzeit-Outcome der Organempfänger zu sichern.

## Fazit für die Praxis

- Tumorscreening ist nicht nur vor- sondern auch nach einer Nierentransplantation (NTX), aufgrund des hohen Risikos einer Tumorentwicklung unter Immunsuppression, essentieller Bestandteil der Patientenbetreuung.
- Urogenitale Tumoren bilden die zweitgrößte Gruppe solider Malignome nach NTX, weshalb ein urologisches Tumorscreening mindestens einmal jährlich für alle Nierenempfänger*innen obligat sein muss.
- Dabei ist besonderes Augenmerk auf die Kontrolle der Eigennieren zu richten, da Nierenzellkarzinome die größte Gruppe der Neoplasien unter diesen Tumoren bilden.
- Patienten mit einer Tumorvorgeschichte, höherem Alter oder Charlson-Score ≥ 4 sollten aufgrund des signifikant erhöhten urogenitalen Tumorrisikos ein individualisiertes Screening erhalten.
- Therapiekonzepte für Hoden- und Prostatakarzinom sowie kurative Therapien des Nierenzell- oder Urothelkarzinoms entsprechen im Wesentlichen denen Nichttransplantierter.
- Für metastasierte Tumoren dieser Entitäten können gerade Immuntherapien nur eingeschränkt Anwendung finden, da diese im Hinblick auf das eklatant hohe Abstoßungsrisiko der Niere in Einzelfallentscheid mit den betroffenen Patienten diskutiert werden müssen.

## Abkürzungen

ASA: American Society of Anesthesiologists
BC: Blasenkarzinom
BMI: Body Mass Index
EBV: Epstein-Barr-Virus
NCC: Nierenzellkarzinom
NTX: Nierentransplantation
NYHA: New York Heart Association
PCa: Prostatakarzinom
PTLD: Posttransplantationslymphom
TC: Hodentumor
TX: Transplantation
